# Multimodal ultrasound muscle assessment in patients with rheumatic diseases: a patient-based reliability study

**DOI:** 10.1093/rap/rkad072

**Published:** 2023-08-18

**Authors:** Gianluca Smerilli, Erica Moscioni, Roberta Sclocco, Maria Giovanna Lommano, Edoardo Cipolletta, Vincenzo Maccarrone, Sonia Farah, Rossella De Angelis, Fausto Salaffi, Walter Grassi, Emilio Filippucci, Andrea Di Matteo

**Affiliations:** Rheumatology Unit, Department of Clinical and Molecular Sciences, Polytechnic University of Marche, ‘Carlo Urbani’ Hospital, Jesi, Ancona, Italy; Rheumatology Unit, Department of Clinical and Molecular Sciences, Polytechnic University of Marche, ‘Carlo Urbani’ Hospital, Jesi, Ancona, Italy; Department of Physical Medicine and Rehabilitation, Spaulding Rehabilitation Hospital, Harvard Medical School, Boston, MA, USA; Rheumatology Unit, Department of Clinical and Molecular Sciences, Polytechnic University of Marche, ‘Carlo Urbani’ Hospital, Jesi, Ancona, Italy; Rheumatology Unit, Department of Clinical and Molecular Sciences, Polytechnic University of Marche, ‘Carlo Urbani’ Hospital, Jesi, Ancona, Italy; Rheumatology Unit, Department of Clinical and Molecular Sciences, Polytechnic University of Marche, ‘Carlo Urbani’ Hospital, Jesi, Ancona, Italy; Rheumatology Unit, Department of Clinical and Molecular Sciences, Polytechnic University of Marche, ‘Carlo Urbani’ Hospital, Jesi, Ancona, Italy; Rheumatology Unit, Department of Clinical and Molecular Sciences, Polytechnic University of Marche, ‘Carlo Urbani’ Hospital, Jesi, Ancona, Italy; Rheumatology Unit, Department of Clinical and Molecular Sciences, Polytechnic University of Marche, ‘Carlo Urbani’ Hospital, Jesi, Ancona, Italy; Rheumatology Unit, Department of Clinical and Molecular Sciences, Polytechnic University of Marche, ‘Carlo Urbani’ Hospital, Jesi, Ancona, Italy; Rheumatology Unit, Department of Clinical and Molecular Sciences, Polytechnic University of Marche, ‘Carlo Urbani’ Hospital, Jesi, Ancona, Italy; Department of Rheumatology, Leeds Institute of Rheumatic and Musculoskeletal Medicine, University of Leeds, Leeds, UK; Department of Rheumatology, National Institute for Health Research, Leeds Biomedical Research Centre, Leeds Teaching Hospitals NHS Trust, Leeds, UK

**Keywords:** sarcopenia, ultrasonography, frailty

## Abstract

**Objectives:**

The aim was to explore the inter-reliability of a newly developed US scanning protocol (multimodal US) for the assessment of different aspects of sarcopenia-related muscle involvement, including muscle mass, muscle quality and muscle stiffness [using point shear-wave elastography (SWE)], in patients with rheumatic and musculoskeletal diseases (RMDs).

**Methods:**

Quadriceps muscle mass (i.e. muscle thickness), muscle quality (i.e. muscle echogenicity evaluated with both a visual semi-quantitative scale and a dedicated software package for image analysis, ImageJ) and point SWE measurements were obtained by two rheumatologists (blinded to each other’s evaluation) in consecutive RMD patients without previous/current myositis or neuromuscular disorders.

Inter-reliability was assessed using the intraclass correlation coefficient (ICC) for continuous variables and Cohen’s kappa (κ) for categorical variables.

**Results:**

A total of 45 RMD patients were enrolled [mean age 54.5 (16.0) years, male-to-female ratio 1:1.5, mean BMI 24.6 (4.6) kg/m^2^], 10 with PsA, 7 RA, 5 AS, 5 PMR, 4 SLE, 4 gout, 4 OA, 3 FM and 3 SSc. The grade of inter-rater reliability was excellent for muscle mass [ICC = 0.969 (0.953 < ICC < 0.979)]. Regarding muscle echogenicity, the agreement was substantial/almost perfect using the visual semi-quantitative scale (weighted linear = 0.793, weighted squared = 0.878) and excellent using ImageJ analysis [ICC = 0.916 (0.876 < ICC < 0.944)]. Finally, a good agreement was obtained for point SWE measurements [ICC = 0.76 (0.712 < ICC < 0.8)].

**Conclusion:**

Multimodal US is a novel and reliable tool for the evaluation of different aspects of muscle involvement (muscle mass, muscle quality and muscle stiffness) in RMD patients.

Key messages

- Multimodal US enables the simultaneous assessment of different aspects of muscle involvement, including muscle mass, muscle quality and muscle stiffness.
- The study showed a good to excellent inter-reliability of multimodal US in RMD patients.
- Multimodal US is a promising and reliable tool for the detection of muscle involvement in RMD patients.

## Introduction

Sarcopenia is characterized by progressive and generalized loss of skeletal muscle mass and strength. It is associated with physical disability, poor quality of life and increased mortality [1]. Sarcopenia is considered primary when no specific cause other than ageing is evident and secondary when predisposing conditions other than ageing, such as autoimmune inflammatory diseases, are present [2].

When secondary to systemic inflammatory diseases, such as RA, sarcopenia might occur at a younger age compared with the general population, as the consequence of pain and decreased mobility, increased production of pro-inflammatory cytokines, and muscle tissue degradation secondary to cellular metabolic alterations [3, 4].

MRI, CT and DXA are regarded as reference imaging tests for the detection of sarcopenia [5, 6]. Their use in clinical practice is limited by feasibility aspects, such as poor availability and costs and, in the case of CT and DXA, also by exposure of the patient to ionizing radiation.

US can depict muscle anatomical details and both quantitative (i.e. decreased muscle mass) and qualitative (i.e. increased muscle echogenicity, which represents fatty and fibrous infiltration) muscle changes [7]. Furthermore, by evaluating muscle stiffness, US shear-wave elastography (SWE) is emerging as a promising tool for the assessment of muscle physiological and biomechanical status [8]. Several studies have shown a good correlation between US measurements and other imaging techniques, such as MRI, CT and DXA, and clinical measures for muscle strength and function (i.e. grip strength, physical performance) [9].

Previous studies have demonstrated the potential value of US in the detection of sarcopenia; most of these studies were carried out in geriatric populations, and the applications of this imaging tool in patients with rheumatic and musculoskeletal diseases (RMDs) have scarcely been investigated [10].

In a recent study, our research group developed a new US scanning protocol for the assessment of multiple aspects of sarcopenia-related muscle involvement, including muscle mass, muscle quality and muscle stiffness (multimodal US) [11]. In this study, the US assessment of muscle echogenicity and stiffness (measured with point SWE) was able to discriminate between SLE patients and healthy subjects [11]. Furthermore, an increased US muscle echogenicity was associated with reduced muscle strength and impaired physical performance in both groups [11].

Besides the promising clinical implications this new US protocol (i.e. early detection of sarcopenia-related muscle involvement in RMD patients), its inter-reliability (defined as the degree of agreement between different operators) remains to be determined. Thus, the aim of this study was to evaluate the inter-reliability of multimodal US for the assessment of muscle involvement (i.e. muscle thickness, muscle echogenicity and muscle stiffness) in RMD patients.

## Methods

### Patients

Consecutive patients attending the outpatient clinic of the Rheumatology Unit of ‘Carlo Urbani’ Hospital, in Jesi (Ancona, Italy) were included. Enrolment of patients started in March 2021 and ended in April 2021. Exclusion criteria were age <18 years, history of myositis or neurodegenerative disorders, and intense physical activity in the week preceding the study enrolment. This study was approved by the local ethics committee, Comitato Etico Regione Marche (CERM no. 155/2021). All the patients gave their informed consent.

### US assessment

Two rheumatologists (A.D.M. and G.S., with 11 and 5 years of experience in musculoskeletal US, respectively) performed a bilateral US assessment of the quadriceps muscle, blinded to each other’s evaluations.

**Figure 1.**
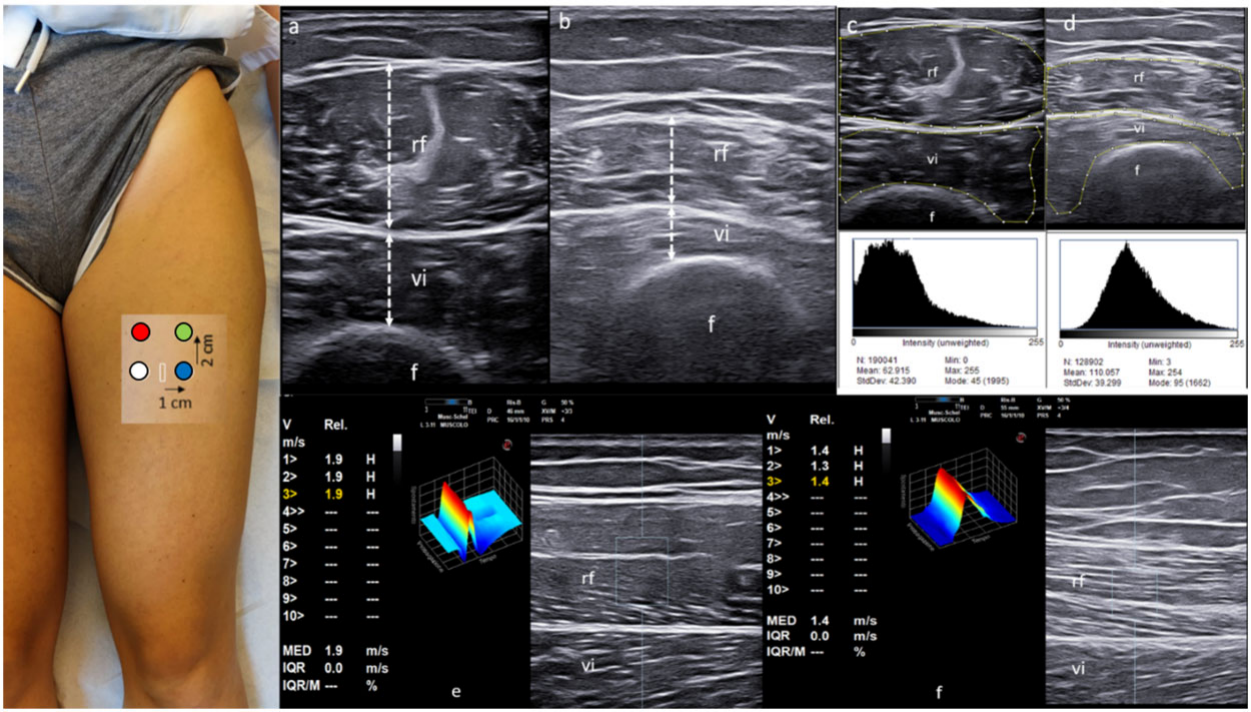
Visual representation of multimodal US. With patients in a relaxed supine position, the midpoint between the anterior superior iliac spine (ASIS) and upper pole of the patella (rectangle) was identified using a measuring tape. (**A**, **B**) Measurement of muscle thickness (dashed lines) of the rectus femoris (rf) and vastus intermedius (vi) muscles (transverse approach). The quadriceps muscle thickening is calculated as the sum of the rf and vi thicknesses. (**A**) A normal muscle in a PsA patient (quadriceps muscle thickness: 45.3 mm), while (**B**) An atrophic muscle in a patient with SSc (quadriceps mass thickness: 18.3 mm). (**C**, **D**) Muscle echogenicity assessment (transverse approach), revealing different grades of muscle echogenicity of the rf and vi muscles in the PsA patient (C) and SSc patient (D) (score=0 and score=3 according to the visual semi-quantitative scale, respectively). Different grades of muscle echogenicity were also obtained with ImageJ assessment (C: mean pixel = 62.9; D: mean pixel = 110.1). The region of interest (ROI) for ImageJ analysis is included within the small squares and lines. (**E**, **F**) Shear-wave elastography (SWE) assessment (longitudinal approach). Starting from the initial anatomical landmark (i.e. the midpoint between the ASIS and upper pole of the patella, white rectangle), SWE measurements were obtained in four distinct muscle areas: 1 cm medial to the muscle central aponeurosis (medial), 1 cm laterally (lateral), 2 cm proximal to medial (upper medial) and 2 cm proximal to lateral (upper lateral). The median of the three measurements for each area was recorded as SWE velocity in units of metres per second, with corresponding interquartile ranges (IQR). The ROI for SWE measurements (rectangle) was placed between the superficial fascia of the rectus femoris muscle and the interface with the vastus intermedius muscle

As previously reported [11], the US scans were carried out using a MyLab X9 (Esaote SpA, Genova, Italy) US machine equipped with a 3–11 MHz linear probe, using the following settings: frequency: 9.0 MHz; gain: 50 dB; depth 5 cm (6 cm in the case of obese patients). A visual representation of the US scanning protocol, which was used to assess muscles in this study, is presented in Fig. 1. Patients lay on the examining table in the supine position, with the legs extended, in a relaxed position. During the US assessment, the following aspects were evaluated.

#### Muscle thickness

Rectus femoris (RF) and vastus intermedius (VI) muscle thicknesses were measured, in addition to their sum (quadriceps muscle thickness), adopting the anterior superior iliac spine (ASIS) technique [12]. This technique consists of the identification of the anterior superior iliac spine and upper pole of the patella by palpation. Then the midpoint between these two structures is identified using a measuring tape and used as a landmark for the acquisition of US transverse scan images.

#### Muscle echogenicity

Echogenicity of the RF and VI muscles was assessed using a modified version of the Heckmatt scale [13]. This modified visual semi-quantitative scale, which was recently developed by our research group, grades muscle echogenicity from zero to three, where 0 = normal (normal hypoechoic muscle), 1 = mild (homogeneously distributed overall increase of the echogenicity involving ≤1/3 of the entire muscle tissue), 2 = moderate (homogeneously distributed overall increase of the echogenicity involving >1/3 but ≤2/3 of the entire muscle tissue) and 3 = severe (homogeneously distributed overall increase of the echogenicity involving >2/3 of the entire muscle tissue) [11, 14]. The muscle echogenicity assessment was performed at the midpoint between the ASIS and the upper pole of the patella using a transverse approach, as described above.

Muscle echogenicity was also determined using ImageJ (v.1.53e) by both US operators. This software, which is a public-domain Java-based image analysis program, measures the greyscale intensity in a region of interest using a histogram function [15]. The RF and VI muscles were selected as the region of interest to determine the mean pixel greyscale intensity. The mean echogenicity was expressed as a value (i.e. mean pixels) between 0 (black) and 255 (white).

#### Muscle stiffness

Measurements of SWE were obtained with the probe oriented longitudinally at four distinct muscle quadrants of the RF muscle (medial, lateral, upper medial and upper lateral) and repeated three times for each muscle area evaluated. Therefore, a total of 24 SWE acquisitions (at four different anatomical sites for each leg) was obtained in the included patients. The quadrants were identified using the midpoint between the ASIS and upper pole of the patella as the starting point. From this midpoint, the probe was translated 1 cm medial to the central aponeurosis (medial), then 1 cm laterally (lateral), then 2 cm proximal to medial (upper medial) and, finally, 2 cm proximal to lateral (upper lateral). There was no predefined order to the sequence for obtaining SWE, but distal measurements (medial and lateral) always had to be obtained before proximal measurements (upper medial and upper lateral). The median of the three measurements for each area was recorded as SWE velocity in units of metres per second, with the corresponding interquartile range. The regions of interest for SWE measurements and depth were placed between the superficial fascia of the RF muscle and the interface with the VI muscle.

### Statistical analysis

Inter-operator reliability for continuous variables (i.e. muscle mass, ImageJ-derived echogenicity and point SWE) was assessed using the intraclass correlation coefficient (ICC), with the 95% confidence interval. For categorical, ordinal variables (i.e. visual semi-quantitative echogenicity scale), weighted Cohen’s kappa (κ) was calculated. Finally, the statistical association between echogenicity measures (i.e. visual semi-quantitative scale and ImageJ-derived measure) was probed using Spearman’s correlation coefficient. All estimations were performed using R and RStudio (R v.4.1.2).

The κ coefficients were interpreted according to Landis and Koch [16] (0: no agreement; 0.1–0.20: slight; 0.21–0.40: fair; 0.41–0.60: moderate; 0.61–0.80: substantial; 0.81–1: almost perfect), with ICC agreement according to Koo and Li [17] (<0.50: poor; between 0.50 and 0.75: moderate; between 0.75 and 0.90: good; >0.90: excellent).

## Results

A total of 45 RMD patients were enrolled consecutively [mean age: 54.5 (16.0) years; male-to-female ratio: 1:1.5; mean BMI: 24.6 (4.6) kg/m^2^]. Of the 45 RMD patients, 10 were diagnosed with PsA, 7 RA, 5 AS, 5 PMR, 4 SLE, 4 gout, 4 OA, 3 FM and 3 SSc. Five of 45 RMD patients were on oral CSs (i.e. ≥5 mg/day of prednisolone equivalents).

The muscle thickness was 17.9 (4.2) mm [mean (s.d. )] for the RF muscle, 15.4 (5.2) mm for the VI muscle, 33.3 (8.2) mm for the quadriceps muscle (RF + VI) (minimum: 12.3 mm; maximum: 52.3 mm). According to the operator with more years of experience in the use of US, muscle echogenicity evaluated using the semi-quantitative visual scale had the following distribution: 16% rated as 0 (normal); 37% rated as 1 (mild); 31% rated as 2 (moderate); and 16% rated as 3 (severe). Muscle echogenicity evaluated using ImageJ analysis was equal to 93.2 (13.8) [mean (s.d. )]. Finally, muscle stiffness, as measured by SWE values for each quadrant, was 1.42 (0.24) m/s (medial), 1.53 (0.27) m/s (lateral), 1.45 (0.28) m/s (upper medial) and 1.53 (0.26) m/s (upper lateral).

The visual semi-quantitative assessment and the ImageJ-derived echogenicity measures for muscle echogenicity showed a significant positive association (Spearman’s ρ = 0.49, *P* < 0.001).

The inter-observer agreement for each muscle parameter is shown in Table 1.

**Table 1.** Inter-observer agreement of muscle thickness, echogenicity (semi-quantitative and ImageJ assessment) and muscle stiffness

		Agreement	
		ICC	
	**Right**		
Muscle mass	Rectus femoris muscle thickness	0.956 (0.938 < ICC < 0.981)	
	Vastus intermedius muscle thickness	0.957 (0.923 < ICC < 0.976)	
	Sum (i.e. quadriceps muscle thickness)	0.965 (0.937 < ICC < 0.981)	
	**Left**		
	Rectus femoris muscle thickness	0.966 (0.938 < ICC < 0.981)	
	Vastus intermedius muscle thickness	0.96 (0.928 < ICC < 0.978)	
	Sum (i.e. quadriceps muscle thickness)	0.971 (0.948 < ICC < 0.984)	
	**Total**		
	Bilateral quadriceps muscles	0.969 (0.953 < ICC < 0.979)	

		**κ**	
	**Visual semi-quantitative scale**	**Weighted (linear)**	**Weighted (squared)**
Muscle quality (muscle echogenicity)	Right quadriceps muscle	0.82	0.892
	Left quadriceps muscle	0.756	0.863
	Bilateral quadriceps muscles	0.793	0.878
	**Grayscale analysis with histograms**	**ICC**	
	Right quadriceps muscle	0.91 (0.842 < ICC < 0.949)	
	Left quadriceps muscle	0.926 (0.869 < ICC < 0.959)	
	Bilateral quadriceps muscles	0.916 (0.876 < ICC < 0.944)	
	**SWE**	**ICC**	
Muscle stiffness	Right (all right quadrants)	0.755 (0.684 < ICC < 0.811)	
	Right upper lateral	0.836 (0.721 < ICC < 0.907)	
	Right upper medial	0.778 (0.63 < ICC < 0.872)	
	Right medial	0.645 (0.436 < ICC < 0.788)	
	Right lateral	0.729 (0.556 < ICC < 0.842)	
	Left (all left quadrants)	0.762 (0.693 < ICC < 0.817)	
	Left upper lateral	0.642 (0.432 < ICC < 0.786)	
	Left upper medial	0.664 (0.462 < ICC < 0.8)	
	Left medial	0.856 (0.753 < ICC < 0.918)	
	Left lateral	0.852 (0.746 < ICC < 0.916)	
	Upper lateral (bilateral)	0.743 (0.633 < ICC < 0.823)	
	Upper medial (bilateral)	0.717 (0.6 < ICC < 0.805)	
	Middle lateral (bilateral)	0.806 (0.719 < ICC < 0.868)	
	Middle medial (bilateral)	0.764 (0.662 < ICC < 0.838)	
	Total agreement (all quadrants bilaterally)	0.76 (0.712 < ICC < 0.8)	

ICC: intra-class correlation; κ: Cohen’s kappa; SWE: shear wave elastography.

The grade of inter-rater reliability was excellent for muscle mass [ICC = 0.969 (0.953 < ICC < 0.979)]. Regarding muscle echogenicity, the agreement was substantial/almost perfect using the visual semi-quantitative scale (weighted linear = 0.793, weighted squared = 0.878, respectively) and excellent using ImageJ [ICC = 0.916 (0.876 < ICC < 0.944)]. Finally, a good agreement was found for point SWE [ICC = 0.76 (0.712 < ICC < 0.8)].

## Discussion

The results of this live (i.e. patient-based) study showed a good to excellent reliability of a recently developed US scanning protocol for the assessment of muscle involvement in RMD patients. This newly developed US scanning protocol enables a combined assessment of different elements of muscle involvement, such as muscle mass, muscle quality and muscle stiffness. All these single elements could potentially be implicated in the pathogenesis of sarcopenia. Therefore, their simultaneous assessment appears to be relevant in patients with RMDs, in which different pathogenetic mechanisms leading to sarcopenia have been hypothesized, in comparison to elderly patients with primary (i.e. age-related) sarcopenia [18]. Interestingly, in our previous study, no difference in muscle mass (i.e. one of the three main criteria to define sarcopenia in elderly people) was observed between SLE patients and healthy subjects [11]. This was arguably attributable to relatively young age of the included populations (mean age of SLE patients: 45 years) compared with elderly sarcopenic patients (i.e. >65 years) and highlights the importance of a comprehensive evaluation of different elements of muscle involvement (i.e. muscle quality and muscle stiffness, other than muscle mass) in RMD patients.

There is a vast literature supporting the high reliability of US muscle mass assessment (i.e. muscle thickness measurement) [19]. The ASIS technique, whose reliability in rheumatic patients was shown in a previous study by our group [12], was used in this study to define the anatomical landmarks (i.e. midpoint between the ASIS and upper pole of the patella by palpation) for the evaluation of muscle echogenicity and SWE measurements.

In our previous study [11], we proposed a novel visual semi-quantitative scale for assessment of muscle echogenicity, which was developed taking as reference the Heckmatt scale [13]. This scale was developed by Heckmatt in 1982 in paediatric patients with neuromuscular disorders and has been the reference method for evaluating muscle echogenicity for several years [13]. Unlike the Heckmatt scale, which evaluates the degree of echo-intensity in a muscle area, our modified visual scale incorporates the extent of the muscular tissue showing an increased echogenicity. Indeed, a homogeneous involvement of muscle structures (i.e. widespread increase in echogenicity) would be expected in sarcopenia, as opposed to a focal increase of muscle echogenicity that is commonly observed in neuromuscular disorders (moth-eaten appearance) and in patients with inflammatory myositis [7]. The reliability of our new semi-quantitative scale for muscle echogenicity was tested recently in a multicentric web-based exercise [14]. In the present live (i.e. patient-based) study, the inter-operator agreement for this visual scale for muscle echogenicity was higher (substantial/almost perfect *vs* moderate, respectively), which is arguably more representative of real life if compared with a remote evaluation of static images and clips.

Finally, SWE has a promising role in the assessment of muscle elasticity, and therefore muscle physiological and biomechanical status, in RMD patients. Methodological issues are regarded as the main limitations to the clinical applicability of SWE [20]. Anisotropy, muscle contraction/relaxation status, tissue heterogeneity owing to myotendinous and aponeurotic structures or blood vessels, probe load and measurement depth are all factors that might potentially influence muscle SWE assessment.

As described in the Methods, point SWE measurements were obtained following a standardized and rigorous protocol, which arguably explains the good reliability results obtained for this assessment, although these reliability results were lower than those obtained for the more standardized (and arguably less subjected to variations) assessment of muscle thickness (i.e. good for SWE and excellent for muscle mass measurement).

In a previous study, our newly developed multimodal US has emerged as a promising screening tool for the detection of sarcopenia-related muscle involvement in patients with SLE and, potentially, in RMD patients [11]. Further studies including a larger number patients are needed to confirm the potential utility of this scanning protocol for the early detection of sarcopenia in RMD patients. In this regard, the present study represents an important methodological step for the potential application of this new scanning protocol in clinical practice and in multicentric studies.

Our study has some limitations. First, the two operators who performed the inter-reliability exercise were also those who developed the multimodal US. The external inter-reliability of this newly developed scanning protocol has yet to be investigated. Second, intra-reliability assessment was not performed. This was a real-world study, which included patients attending the outpatient clinic of our Rheumatology unit, and it was not possible to perform a repeated assessment on the same day (or on different days).

### Conclusions

Our recently developed multimodal US can reliably assess different aspects of muscle involvement (muscle mass, muscle quality and muscle stiffness) in RMD patients.

## Data Availability

The data underlying this article will be shared upon reasonable request to the corresponding author.
